# Febuxostat protects from Doxorubicin induced hepatotoxicity in rats via regulation of NF-κB p65/NLRP3 inflammasome and SIRT-1/AMPK pathways

**DOI:** 10.1007/s00210-025-03808-6

**Published:** 2025-02-14

**Authors:** Ahmed M. El-Dessouki, Eman H. Yousef, Nahed A. Raslan, Asmaa I. Alwakeel, Samar Ibrahim, Amany A. Alzokaky

**Affiliations:** 1 Pharmacology and Toxicology Department, Faculty of Pharmacy, Ahram Canadian University, Giza, 12566 Egypt; 2 Pharmacology and Biochemistry Department, Faculty of Pharmacy, Horus University-Egypt, New Damietta, 34518 Egypt; 3 Pharmacology and Toxicology Department, Faculty of Pharmacy (Girls), Al-Azhar University, Cairo, 11651 Egypt; 4 Clinical Pharmacy Program, College of Health Sciences and Nursing, Al-Rayan Colleges, Madina, Saudi Arabia; 5 Pharmacy Practice and Clinical Pharmacy Department, Faculty of Pharmacy, Galala University, Ataka, Egypt

**Keywords:** Febuxostat, Doxorubicin, Hepatotoxicity, SIRT-1/AMPK pathways

## Abstract

Doxorubicin (DOX) is a highly potent broad-spectrum anticancer drug, but it has severe side effects, including hepatotoxicity. Therefore, we evaluated the efficacy of febuxostat (FBX), a specific inhibitor of xanthine oxidase and antioxidant, in blocking hepatotoxicity associated with DOX in rats. Rats were treated with FBX (10 or 15 mg/kg/day orally for 2 weeks) and given DOX (15 mg/kg as single dose at the 7th day, intraperitoneal) to induce hepatotoxicity. The results indicated that FBX could reduce the pathological alterations of liver tissues induced by DOX and ameliorate the inappropriate changes in liver function biomarkers (AST, ALT, and ALP) in serum, oxidative stress parameters (catalase, superoxide dismutase, NOX1, NQO-1, HO-1, Keap-1, and Nrf2) and inflammatory markers in the liver (NF-κB p65, TNF-α, NLRP3). Additionally, FBX attenuated the p53, BAX, cytochrome C, caspase-9, and caspase-3 levels to restrain cell apoptosis. In addition, FBX therapy was found to increase protein levels of SIRT-1 and AMPK in the liver. These findings demonstrate that FBX can reduce the hepatotoxicity caused by DOX in rats through mechanisms that counteract oxidative stress, inflammation, and apoptosis.

## Introduction

The liver is particularly vulnerable to injury caused by chemical exposure as doxorubicin, that accumulates largely in liver. The range of pathological hepatic injuries varies from minor, nonspecific changes in the structure and function of liver to more severe injury such as fibrosis and even the development of liver cancer (Gu and Manautou 2012; Bartlett et al. 2017). Doxorubicin (DOX) is a frontline chemotherapeutic agent employed for the treatment of solid tumor and hematological cancers (Smuder 2019; Montalvo et al. 2021). Upon administration, DOX is quickly eliminated from the bloodstream and favorably accumulates in the liver and kidneys (Pugazhendhi et al. 2018). The cytotoxic effects of DOX are mediated by destabilizing DNA, interfering with replication and transcription processes, and generating oxygen free radicals, which induce cellular damage (Wallace et al. 2020). Despite its notable antineoplastic efficacy, the clinical utility of DOX is constrained due to its propensity to cause toxicity in multiple organs. Although the precise mechanisms underlying DOX-induced hepatotoxicity yet unclear, it is thought that the accumulation and metabolism of DOX in these organs lead to the generation of free radicals, lipid peroxidation, and apoptosis (Ikewuchi et al. 2021; Timm et al. 2021). The non-specific toxicity of DOX demands a systemic therapy that can preserve its antitumor efficiency while mitigating its deleterious effects. Still, there are no approved clinical treatments available to prevent or treat DOX-induced liver toxicity. In this issue, modifying the cytotoxic signaling of DOX to attenuate oxidative damage and inflammation has been proposed as a potential approach (Smuder 2019).

Sirtuin 1 (Sirt1), a NAD^+^-dependent class III histone deacetylase, is essential for numerous cellular and physiological processes, including mitochondrial biogenesis, cell injury, and ferroptosis (Li et al. 2021). Histone deacetylase activity of SIRT1 has been reported to exert a protective mechanism against numerous diseases as pancreatic ductal adenocarcinoma (PDAC) and hepatic injury in which oxidative stress is a major factor (Pan et al. 2022). In liver, Sirt1 activity has been proven to play a critical role in the fight against several damage types, including alcohol-induced liver injury, CCl4-induced liver fibrosis, and hepatic ischemia-reperfusion (I/R) injury (You et al. 2015; Nakamura et al. 2017; Li et al. 2018). Simultaneously, inhibiting SIRT1 expression has been demonstrated to directly affect the Keap1/Nrf2/ARE pathway, which consecutively hinders antioxidant defense systems and raises the reactive oxygen species (ROS) production (Huang et al. 2017). Noteworthy, a recent study showed that Sitr1 overexpression can mitigate the hepatotoxicity caused by DOX by lowering oxidative stress, inflammation, and apoptosis (Xi et al. 2023).

Febuxostat (FBX) is a specific inhibitor of xanthine oxidase that is essential for the management of multiple inflammatory disorders. These inhibitors possess properties such as anti-inflammatory, antioxidant, and immune-modulatory effects. Previous research studies have demonstrated the anti-inflammatory capabilities of FBX using various experimental models (Fahmi et al. 2016). In renal ischemia-reperfusion injury model, FBX effectively suppressed the generation of ROS and exerted antioxidative stress effects (Tsuda et al. 2012). Based on these findings, xanthine oxidase inhibitors offer a promising therapeutic strategy for treating inflammatory liver diseases.

The purpose of this work is to explore the potential role of FBX to attenuate the DOX induced hepatoxicity targeting cascade of inflammatory, oxidative stress and apoptosis pathways.

## Materials and methods

### Docking of febuxostat to Sirt1

The crystal structures of Sirt1 (PDB ID: 4ZZJ and 4ZZH) was downloaded from the Protein Data Bank (https://www.rcsb.org/pdb/home/home.do). In protein, water molecules and complexes bound to the protein were deleted. Moreover, amino acid residues ionization and tautomeric states were adjusted by adding hydrogens to the protein. The binding active site is identified based on the site of cocrystallized ligand (4TQ). The ZINC database was used to extract the three-dimensional configuration of FBX, and its chemical structure optimization was carried out by modifying the bond order, adding charges, and finally adding hydrogens. Additionally, energy minimization by conformational search or at least clean up geometry was applied. Before adding hydrogens, bond order and charges were adjusted. The PDBQT file format was used to enhance readability of the protein and ligand structure in the AutoDock program. Molecular docking was carried out in cocrystallized ligand binding site using AutoDock vina. The docking estimations were established in relation to the X, Y, Z dimensions in the used grid map as indicated −0.423893, 44.524196 and −0.038714 for 4ZZJ and 25.921661, −53.737000 and 2.384964 for 4ZZH, respectively. The binding mode and interactions in the binding pocket of the obtained poses were visualized and analyzed using Chimera 1.15 and Discovery Studio Visualizer v21.1.0.20298.

### Animals and treatment

Adult male Wistar rats (200 ± 20 g) were obtained from the National Institute for Research, Cairo, Egypt. The animals were housed under control conditions in terms of constant temperature (23 ± 1 °C), humidity (60 ± 10%), and a 12/12 h light/dark cycle. They were allowed standard rat chow and water ad libitum. The experimental procedures started one week later to allow animal adaptation. In this study, the used experimental protocol was approved by the Animal Ethics Committee (No. REC0324) of the Faculty of Pharmacy, Al-Ahram Canadian University, Egypt, in compliance with “Principles of Laboratory Animal Care” (NIH publication No. 85-23, revised 1985) in animal care and experiments.

DOX (Adriamycin vial, 2 mg/mL) was bought from Hikma Pharmaceuticals (Cairo, Egypt). FBX was purchased from Eva pharma company (Cairo, Egypt), FBX was prepared for orally administration by dissolution in 1% Tween 80. The formalin solution was bought from Merck Co. (Darmstadt, Germany). All additional chemicals required for the experiments were bought from Sigma-Aldrich Co. (Munich, Germany).

### Experimental design

**Fig. 1:**
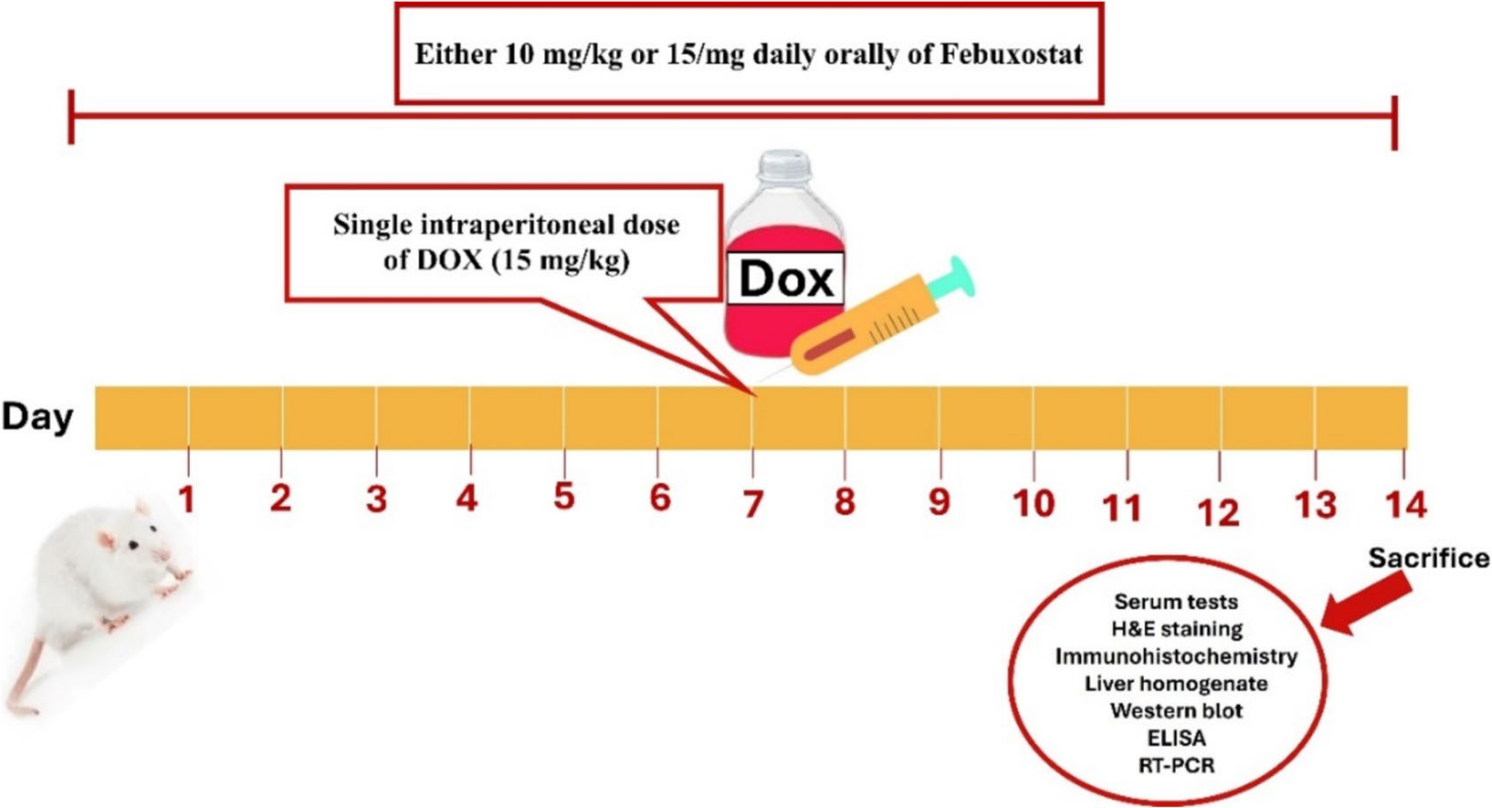
Schematic diagram illustrates the experimental design. Rats received either 10 or 15 mg/kg FBX orally for 14 days. After 15 days, The DOX induced hepatotoxicity model was established by injecting 15 mg/kg of DOX i.p. as single dose. The blood and liver were harvested for various experimental methods

Rats were distributed into four groups (6 rats in each group). As a control group, the first group administered vehicle only, the second group administered single intraperitoneal dose of DOX (15 mg/kg) on 7th day, the third group administered combination of a low dose of FBX (10 mg/kg; P.O.) for 14 days and DOX (15 mg/kg, I.P) on 7th day of treatment, the forth group administered combination of a high dose of FBX (15 mg/kg; P.O.) for 14 days and DOX (15 mg/kg, I.P) on 7th day of treatment. The FBX regimen was initiated one week prior to the DOX injection and was administered daily for a duration of 14 days as shown in Fig. 1.

On day 14, prior to sacrifice, rats were weighted and anesthetized with pentobarbital sodium (40 mg/kg, i.p.). Blood samples were taken from the retro‐orbital vein of the eye and centrifugated at 4000 rpm for 5 min to obtain serum, which was afterward stored at –80 ^◦^C. The liver was rapidly removed, weighed, cleaned and homogenized in 10 mM ice-cold Tris-HCl buffer (pH 7.4). After the centrifugation of homogenate, the clear supernatant was removed and stored at − 80 ^◦^C. Portion of liver samples were maintained in 10% neutral buffered formalin (NBF) for histological evaluation.

### Biochemical assay

#### Enzyme-linked immunosorbent assays (ELISA) markers

##### Liver function markers

For hepatic toxicity, serum alkaline phosphatase (ALP) (Cat No. E4575-100), alanine aminotransferase (ALT) (Cat No E4325-100) and aspartate aminotransferase (AST) (Cat No. E4321-100) were measured using ELISA kit as stated by the recommendation of the manufacturer (Biovision, Milpitas, USA).

##### Oxidative stress markers

The liver homogenate was examined for level of Catalase (CAT) (Cat No. MBS2600683), superoxide dismutase (SOD) (Cat No. CSB-E08555r) as markers of lipid peroxidation (LPO), heme oxygenase-1 (HO-1) (Cat. No. E-EL-R0488) and NAD(P)H quinone oxidoreductase 1 (NQO-1) (Cat. No. MBS7606601) based on the kits instructions purchased from MyBioSource, Cusabio Biotech Co., Ltd, Elabscience, MyBioSource, respectively.

##### Inflammatory markers

Nuclear factor-erythroid 2-related factor 2 (Nrf2) content in liver homogenate was detected; Cat No. E1083Ra, Keap1; Cat No. LS-F56169, TNF-α; Cat No. CSB-E11987r and NLRP3; Cat No. RK04262 as markers of inflammation based on the kits instructions purchased from bioassay technology laboratory, LSBIO, Cusabio Biotech Co., Ltd and ABclonal technology respectively.

##### Apoptotic markers

Caspase 3 content in liver homogenate was detected: Cat No. E1648Ra, cytochrome C (Cyt. C); Cat No. CSB-EL006328RA and BAX; Cat No. CSB-EL002573RA as markers of apoptosis following the instructions on the reagent kits obtained from bioassay technology laboratory and Cusabio Biotech Co., Ltd respectively.

#### Real time PCR

Real-time polymerase chain reaction (RT-PCR) was used to measure the expression of NOX1, AMPK, P53, and Sirt1 mRNA. Firstly, TRIzol reagent (Invitrogen Corporation, Grand Island, NY, USA) was used to extract total RNA from liver tissues in accordance with the manufacturer's instructions. Then, the purity of RNA was proved spectrophotometrically at 260/280 nm. Afterthat, high-capacity cDNA Reverse Transcription Kit (Applied Biosystems, Foster City, CA) was used to convert equal amounts of RNA (2 µg) into cDNA. Eventually, PCR amplification was carried out in 20 µl reaction mixture consisting of 10 µl SYBR® Green PCR Master Mix (Applied Biosystems, Foster City, CA), 1 µl forward primer (nM), 1 µl reverse primer (nM), 2 µl cDNA, and 6 µl water using Step One thermal cycler. All the used primers were synthesized by Invitrogen and their sequences were listed in Table 1. β-actin was used as a housekeeping gene and the expression of mRNA levels in each sample was normalized to its mRNA levels. The relative expression was calculated according to the 2^−ΔΔCT^ formula (Livak and Schmittgen 2001).

**Table 1:** Primers used for qRT-PCR

Gene	Description	Primer Sequence	Reference Sequence	Product size
NOX1	F	5′-TAAGAGGCTCCAGACCTCCAT −3′	NM_053683.2	299
	R	5′- CTCAGCGTGTGGTTGCAAAA-3′		
AMPK	F	5′-CATTCTTGGTTGCCGAAACA-3′	NM_023991.1	70
	R	5′-TGTTTGGATTTCTGTGGGTT-3′		
P53	F	5′- GTTCCGAGAGCTGAATGAGG-3′	NM_030989.3	125
	R	5′-TTTTATGGCGGGACGTAGAC-3′		
Sirt1	F	5′-CGCCTTATCCTCTAGTTCCTGTG-3′	NM_001372090.1	136
	R	5′-CGGTCTGTCAGCATCATCTTCC-3′		
β-actin	F	5′-TCCTCCTGAGCGCAAGTACTCT-3′	NM_031144.3	153
	R	5′-GCTCAGTAACAGTCCGCCTAGAA-3′		

*F*, forward; *R*, reverse

#### Histopathological and immunohistochemical examination

Some liver tissues were excised and promptly fixed in 10% neutral buffered formalin, dehydrated in ascending grades of ethanol, cleared in xylene and embedded in paraffin. 3 μm thickness sections were cut and stained with haematoxylin and eosin (H&E). To prevent bias, all histopathologic processing and specimen evaluation were blindly carried out by an experienced observer.

5-μm deparaffinized liver sections were used for immunohistochemical studies. The sections were treated with monoclonal antibodies against nuclear factor kappa B p65 (NF-κB p65) (Cat. No. ab131100; Abcam Co.) and caspase 9 (Cat. No. A11910; ABclonal Co.) for whole night at 4˚C. Then, secondary antibodies (Abcam) coupled to horseradish peroxidase were added to the sections. 2% diaminobenzidine reagent in 50 mM Trisbuffer, pH 7.6, was applied for visualization. The immunohistochemically stained slides were photographed a light microscope (Olympus CH2). The immunostained regions for Caspase-9 and NF-κB p65 were identified in a blind manner using ImageJ software (National Institutes of Health, Bethesda, MD, USA at 400 × magnification. For digital image analysis, size of all images was adjusted to 12.7 cm in width and 9 cm in length (300 dpi) and a scale bar was set to (25-μm). Finally, all images were loaded into the (image J) program and percentage of positive area was calculated.

#### Statistical analysis

GraphPad prism 9.5.1 statistical software was used to analyze data. Statistical analysis was conducted using one way ANOVA followed by Tukey-Kramer as a post-hoc test, data were represented as means ± SE and *P* < 0.05 was used to indicate statistical significance.

## Results

### FBX molecular interaction study

**Fig. 2:**
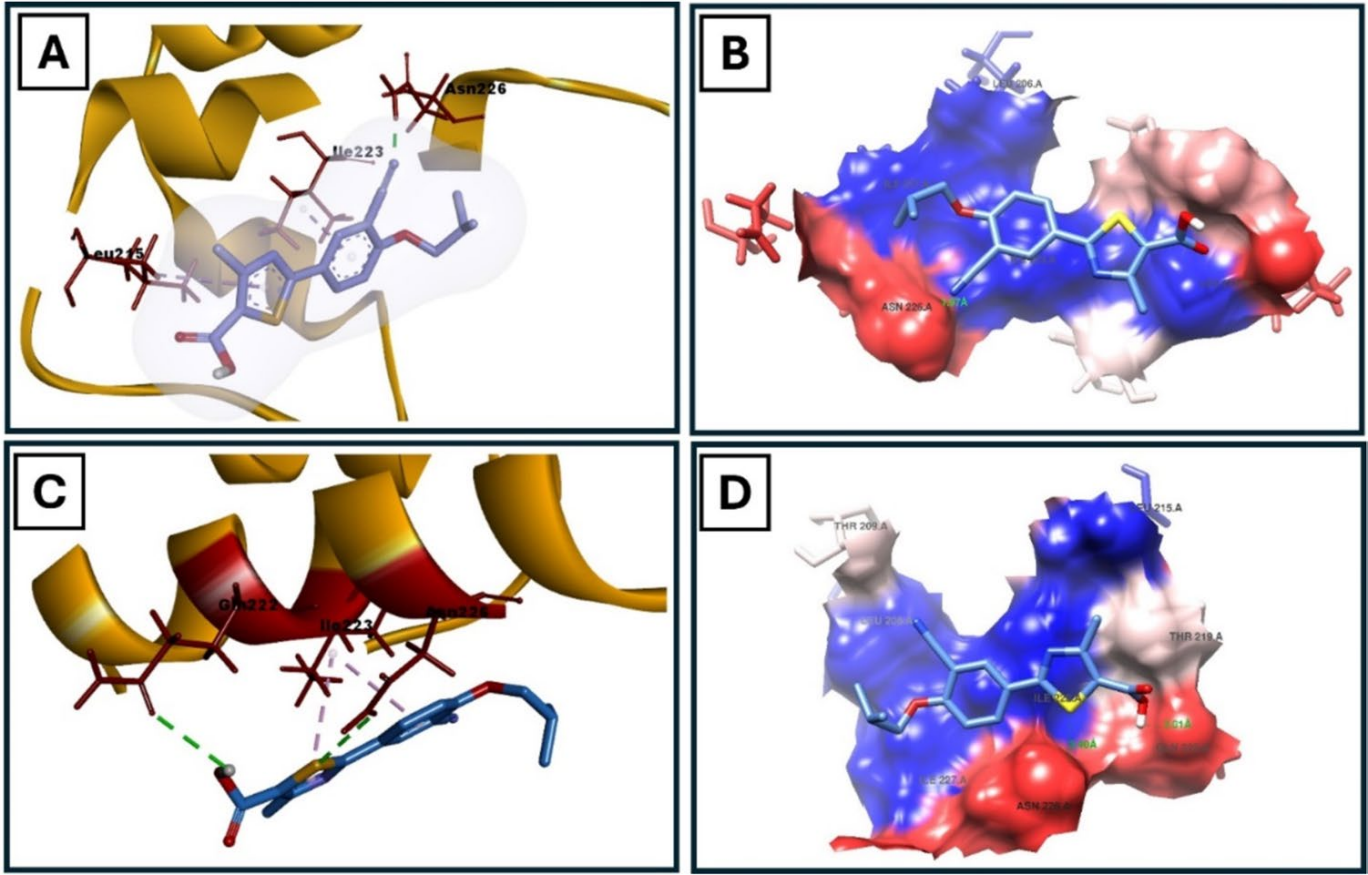
Docked pose of FBX in the binding pocket of Sirt1. **A** Participating amino acids in the interaction of Sirt1 (PDB code: 4ZZJ) using Discovery Studio Visualizer. **B** Hydrophobic interactions between FBX and Sirt1 pocket (PDB code: 4ZZJ) using Chimera. **D** Participating amino acids in the interaction of Sirt1 (PDB code: 4ZZH) using Discovery Studio Visualizer. (F) Hydrophobic interactions between FBX and Sirt1 pocket (PDB code: 4ZZH) using Chimera

FBX/Sirt1 binding interactions were estimated using Autodock Vina 1.1.2 (Trott and Olson 2010). The binding affinity was −3.9 kcal/mol when FBX interacted with the Sirt1 in the site of 4TQ (PDB code: 4ZZJ). A hydrogen bonds was established between FBX with ASN226 of Sirt1. The binding of FBX with Sirt1 is promoted by hydrophobic interactions with Leu206, ile227, ile223 and leu215. The binding affinity was −3.7 kcal/mol when FBX interacted with the Sirt1 in the site of 4TQ (PDB code: 4ZZH). A two hydrogen bonds was established between FBX with GLN222 and ASN226 of Sirt1. The binding of FBX with Sirt1 is promoted by numerous hydrophobic interactions with LEU206, LEU215, ILE227 and ILE223. The docking results were summarized in Fig. 2 and Table 2.

**Table 2:**
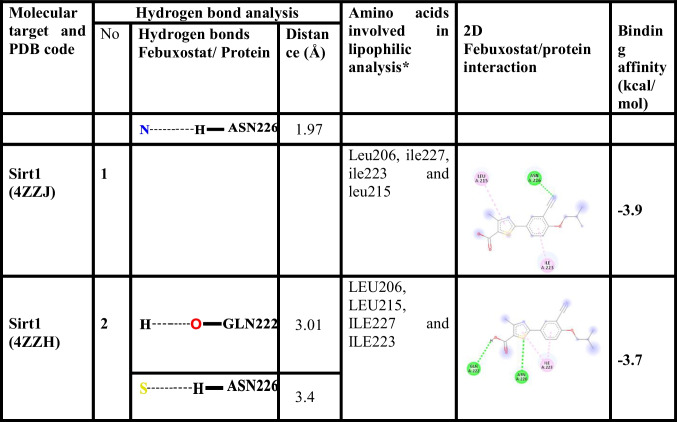
Interaction type, amino acids and the binding affinity involved in interaction of Sirt1 with FBX

### FBX ameliorates liver function in DOX-administered rats

**Fig. 3:**
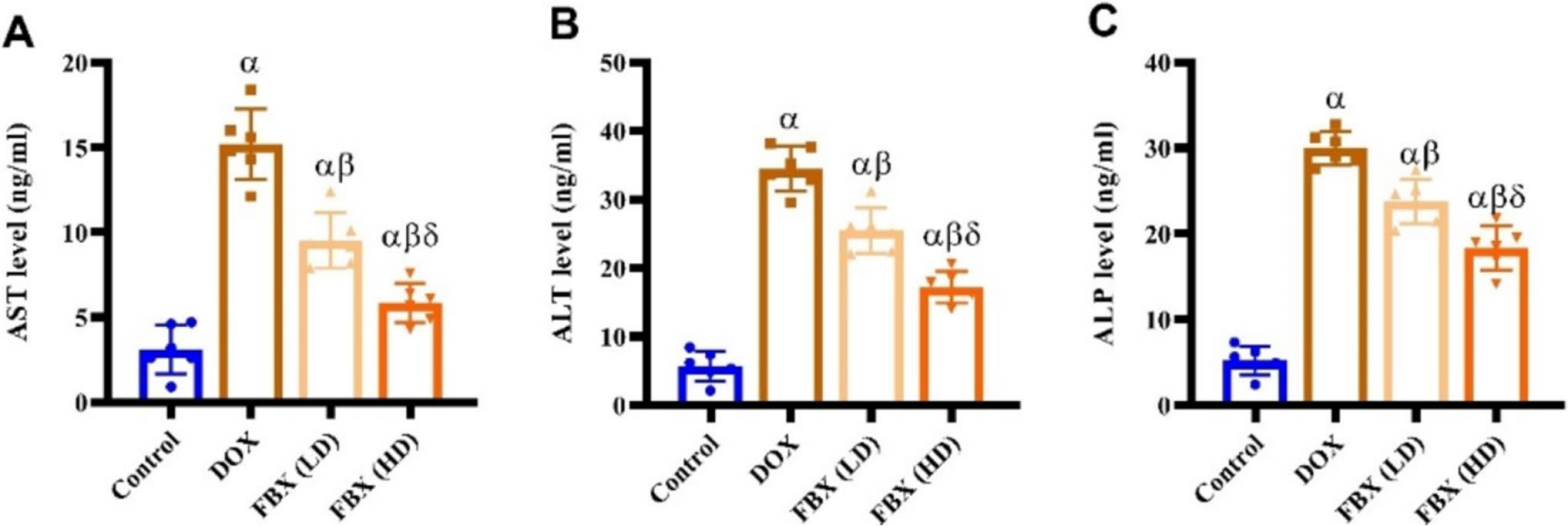
FBX ameliorates liver function in DOX-administered rats. Effect of FBX in low and high doses on **A** AST, **B** ALT, and **C** ALP**.** Data are shown as mean ± SE, (*n* = 6). α, β, δ indicate significance between the control group, DOX group and DOX + FBX. (LD) treated group respectively using ONE WAY ANOVA test at a *P*-value < 0.05 followed by Tukey

To assess the impact of DOX on liver function, the serum concentrations of AST, ALT, and ALP were measured. DOX administration (15 mg/kg, I.P.) markedly impaired liver function, evidenced by significant increases in the levels of AST, ALT, and ALP, which were elevated to 3.9 folds, 5.03 folds, and 4.78 folds respectively, in relation to the baseline levels of the control group. On the other hand, liver function showed considerable improvement in the group that received pre-treatment with FBX. (10 mg/kg), with AST, ALT, and ALP levels reducing by 0.37 folds, 0.26 folds and 0.21 folds respectively, in relation to the DOX-treated group. Additionally, more pronounced improvements were observed with FBX. pre-treatment at a dose of 15 mg/kg, where AST, ALT, and ALP levels decreased by 0.62 folds, 0.50 folds and 0.39 folds respectively, against the DOX-treated group. Furthermore, in relation to the group pre-treated with FBX. (10 mg/kg), the group pre-treated with FBX. (15 mg/kg) demonstrated significant further reductions in AST, ALT, and ALP levels by 0.38 folds 0.32 folds, and 0.23 folds respectively, as depicted in Fig. 3A-C.

**Fig. 4:**
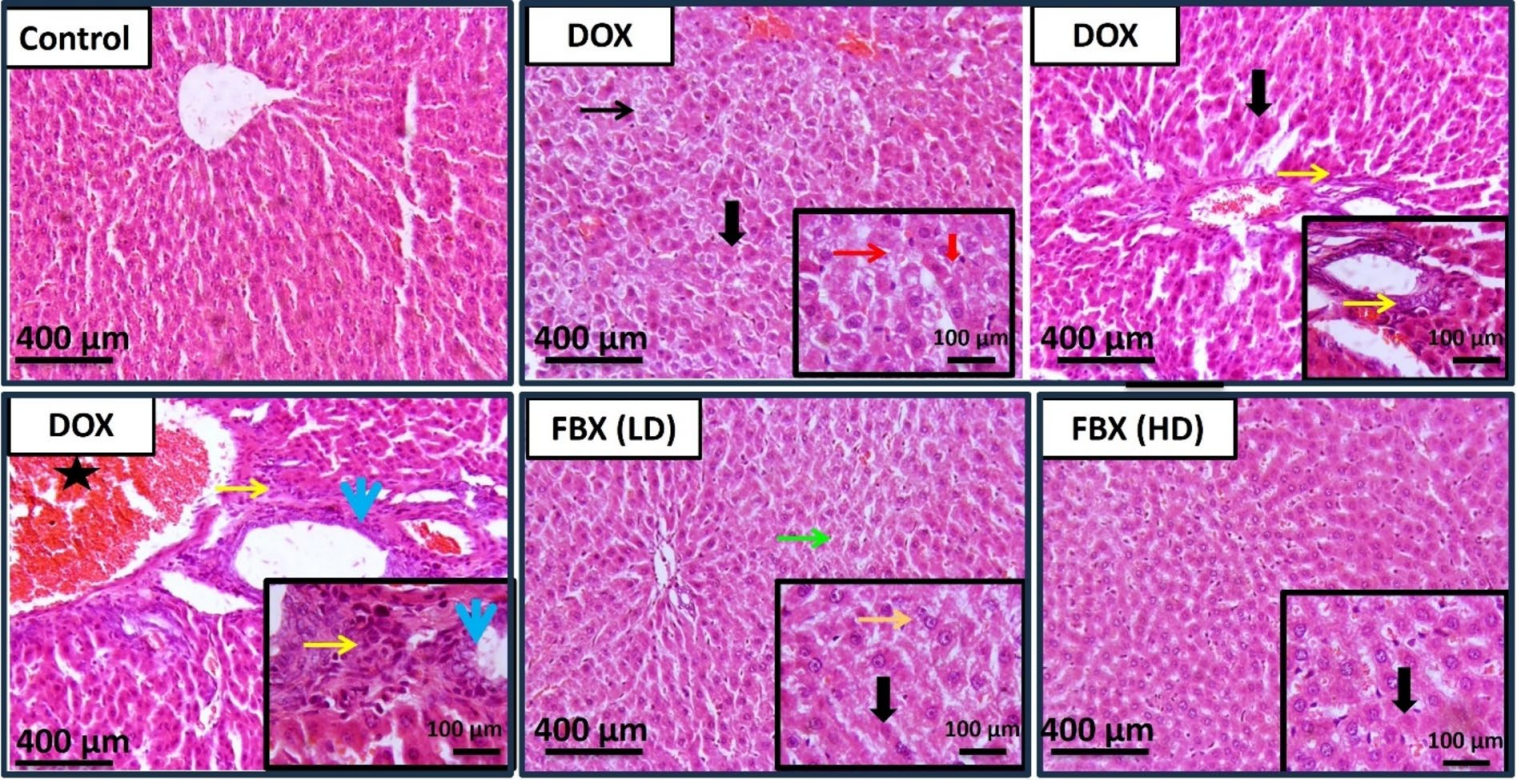
FBX Attenuated DOX-induced liver histopathological changes. The effect of FBX on DOX-Induced liver histopathological changes was assessed in different groups by histopathological examination of hepatic tissue stained by H&E. Thin black arrow represents diffuse hepatocellular degeneration, thick black arrows represent necrosis, red thin arrow represents diffuse swollen hepatocytes, red thick arrow represents hyperesinophilic hepatocytes with pykontic nuclei, yellow thin arrows represent periportal fibrosis admixed with few inflammatory cells and reactive bile duct lined with 2 layer of cells, thin blue arrows represent bile duct reactions with periductal fibrosis and inflammation, star represents extensive portal congestion, thin green arrow represents minimal to mild hepatocellular degeneration and thin orange arrow represents swollen hepatocytes. Image magnification = × 100, inset = × 400

H&E-stained hepatic sections (Fig. 4) of control group revealed normal histological appearance of hepatocytes. Meanwhile, the hepatic sections of DOX group showed a marked degeneration, necrosis, periportal fibrosis admixed with inflammatory cells and extensive portal congestion. FBX (LD) group showed minimal to mild hepatocellular degeneration. Also, FBX (HD) group showed normal histological appearance of hepatocytes with occasional individual cell necrosis.

### FBX ameliorates oxidative stress markers in DOX-administered rats

#### Catalase, SOD activity and NOX1 expression

**Fig. 5:**
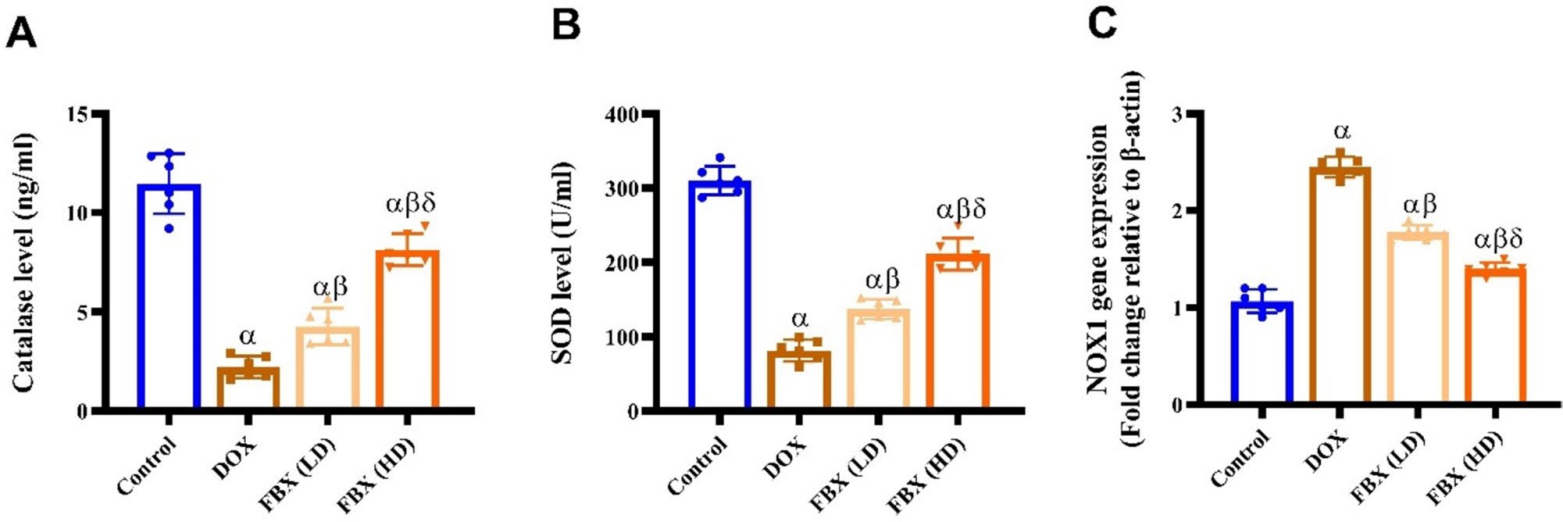
FBX ameliorates oxidative stress markers in DOX-administered rats. Effect of FBX in low and high doses on **A** Catalase level, **B** SOD level, and **C** NOX1 expression. Data are shown as mean ± SE, (*n* = 6). α, β, δ indicate significance between the control group, DOX group and DOX + FBX. (LD) treated group respectively using ONE WAY ANOVA test at a *P*-value < 0.05 followed by Tukey

As shown in Fig. 5A–C activity of catalase and SOD was significantly decreased (*p* < 0.05) in DOX group by 0.8 folds and 0.74 folds compared with control while NOX1 gene expression was significantly increased (*p* < 0.05) in DOX group by 1.27 folds compared with control group. However, pretreatment with FBX. in low and high doses significantly increased (*p* < 0.05) catalase activity by 0.93 and 2.68 folds and SOD activity by 0.68 and 1.59 folds respectively compared with DOX group, decreased (*p* < 0.05) NOX1 gene expression by 0.27 and 0.43 folds respectively compared with DOX group. Furthermore, in relation to the group pre-treated with FBX. (LD10 mg/kg), the group pre-treated with FBX. (HD 15 mg/kg) demonstrated significant further elevation in catalase, SOD by 0.9 folds 0.54 folds and decrease in NOX1 levels by 0.21 folds respectively.

#### SIRT-1, NQO-1, HO-1, Keap-1 and Nrf2 content

**Fig. 6:**
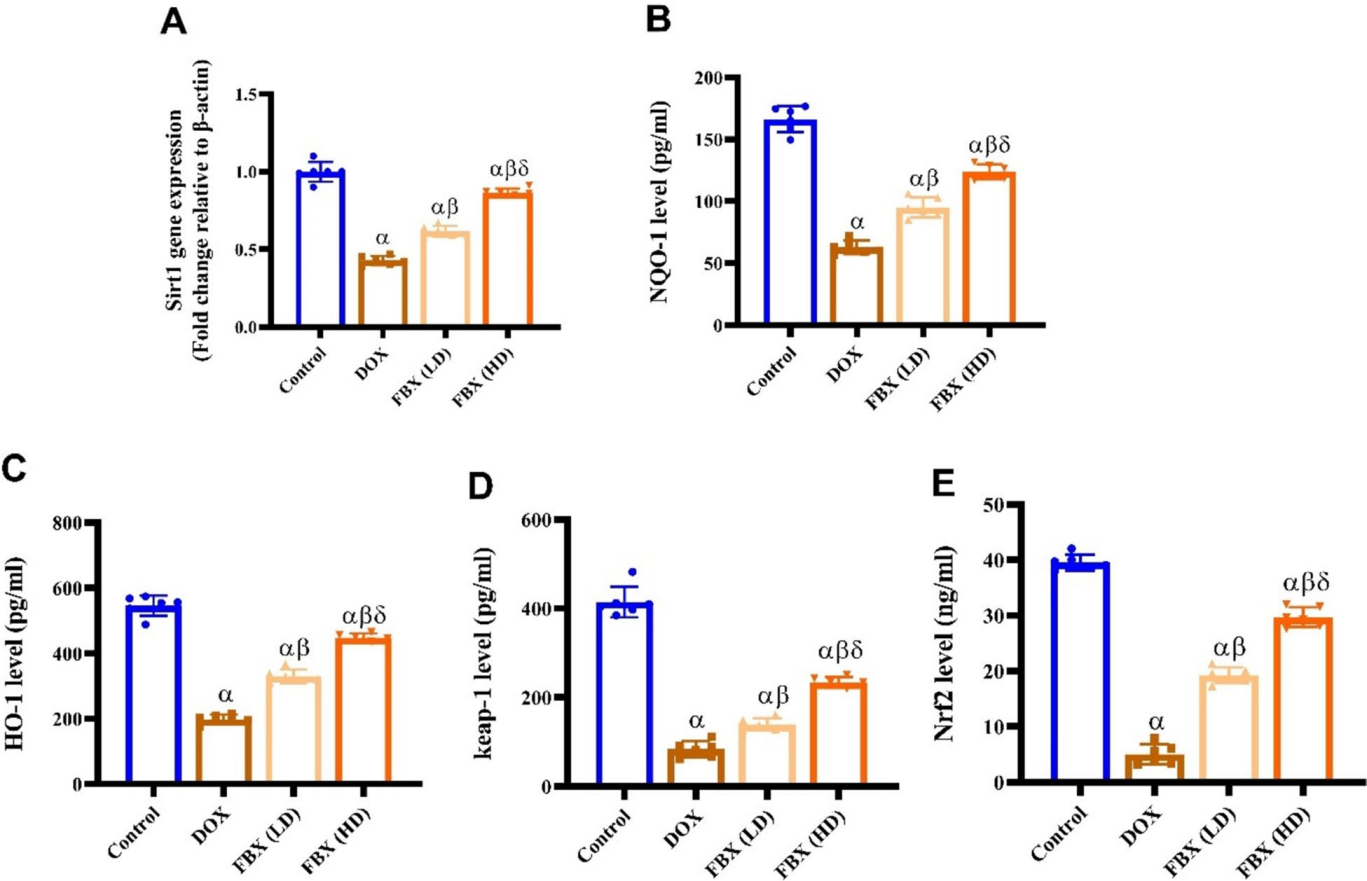
FBX exerts antioxidant activity in DOX-administered rats. Effect of FBX in low and high doses on **A** SIRT-1, **B** NQO-1, **C** HO-1, **D** Keap1 and **E** Nrf2. Data are shown as mean ± SE, (*n* = 6). α, β, δ indicate significance between the control group, DOX group and DOX + FBX. (LD) treated group respectively using ONE WAY ANOVA test at a *P*-value < 0.05 followed by Tukey

As shown in Fig. 6A–C content of SIRT-1, NQO-1 and HO-1 was significantly decreased (*p* < 0.05) in DOX group by 0.58, 0.62 and 0.63 folds compared with control group, respectively. However, pretreatment with FBX. in low and high doses significantly increased (*p* < 0.05) SIRT-1content by 0.44 and 1.02 folds, NQO-1content by 0.51 and 0.97 folds and HO-1 content by 0.66 and 1.25 folds respectively compared with DOX group. In Fig. 6D, E, content of Keap-1 and Nrf2 was significantly decreased (*p* < 0.05) in DOX group by 0.79 folds and 0.87 folds compared with control group. However, pretreatment with FBX. in low and high doses significantly increased Keap-1 content by 0.66 and 1.79 folds and increased (*p* < 0.05) Nrf2 content by 2.83 and 4.89 folds respectively compared with DOX group. Furthermore, in relation to the group pre-treated with FBX. (LD10 mg/kg), the group pre-treated with FBX. (HD 15 mg/kg) demonstrated significant further elevation in SIRT-1, NQO-1, HO-1, Keap-1 and Nrf2 content by 0.4, 0.3, 0.35,0.68 and 0.54-folds respectively.

### FBX ameliorates inflammatory markers in DOX-administered rats

**Fig. 7:**
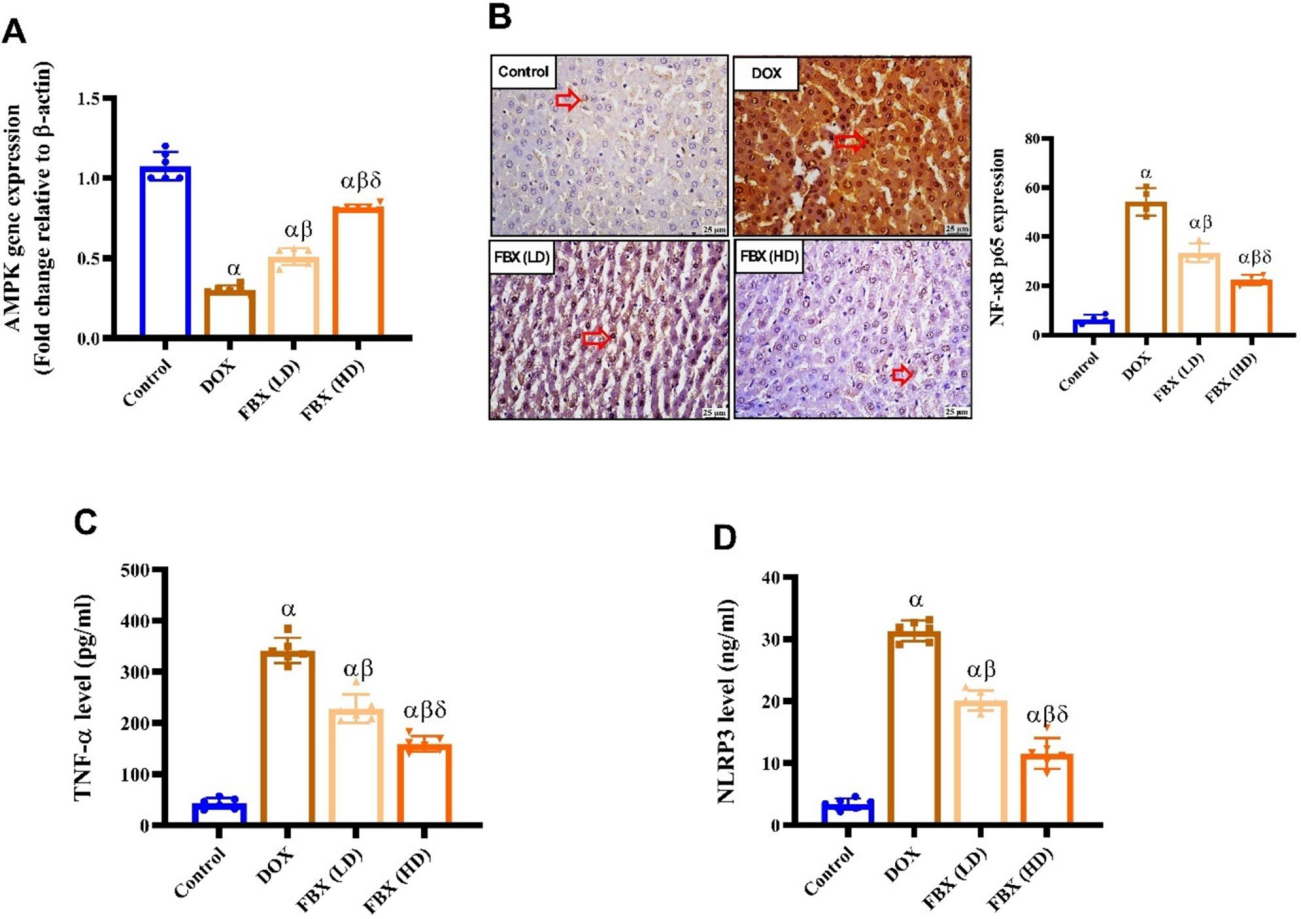
FBX ameliorates inflammatory markers in DOX-administered rats. Effect of FBX in low and high doses on **A** AMPK, **B** NF-κB, **C** TNF-α and **D** NLRP3. Red arrows point to positive expression. IHC counterstained with Mayer's hematoxylin (X400). Data are shown as mean ± SE, (*n* = 4). α, β, δ indicate significance between the control group, DOX group and FBX (LD) treated group respectively using ONE WAY ANOVA test at a P-value < 0.05 followed by Tukey

AMPK gene expression was significantly decreased (*p* < 0.05) in DOX group by 0.72-fold compared with control group. Pretreatment with FBX. in low and high doses significantly increased (*p* < 0.05) AMPK gene expression by 0.68 and 1.69 folds respectively compared with DOX group while expression of NF-κB p65 significantly increased (*p* < 0.05) in DOX group by 7.45 folds compared with control group. pretreatment with FBX. in low and high doses significantly decreased (*p* < 0.05) NF-κB p65 expression by 0.38 and 0.59 folds. Content of TNF-α & NLRP3 were significantly increased (*p* < 0.05) in DOX group by 7.8 and 8.15 folds compared with control group. pretreatment with FBX. in low and high doses significantly decreased (*p* < 0.05) TNF-α content by 0.33 and 0.53 folds & NLRP3 by 0.35- and 0.63-folds content respectively compared with DOX group. Furthermore, in relation to the group pre-treated with FBX (LD10 mg/kg), the group pre-treated with FBX (HD 15 mg/kg) demonstrated significant further elevation in AMPK by 0.6 folds and decrease in NF-κB p65 expression, TNF-α & NLRP3 content by 0.33, 0.3 and 0.43 folds as shown in Fig. 7A-D.

### FBX attenuates apoptosis cascades in DOX-administered rats

**Fig. 8:**
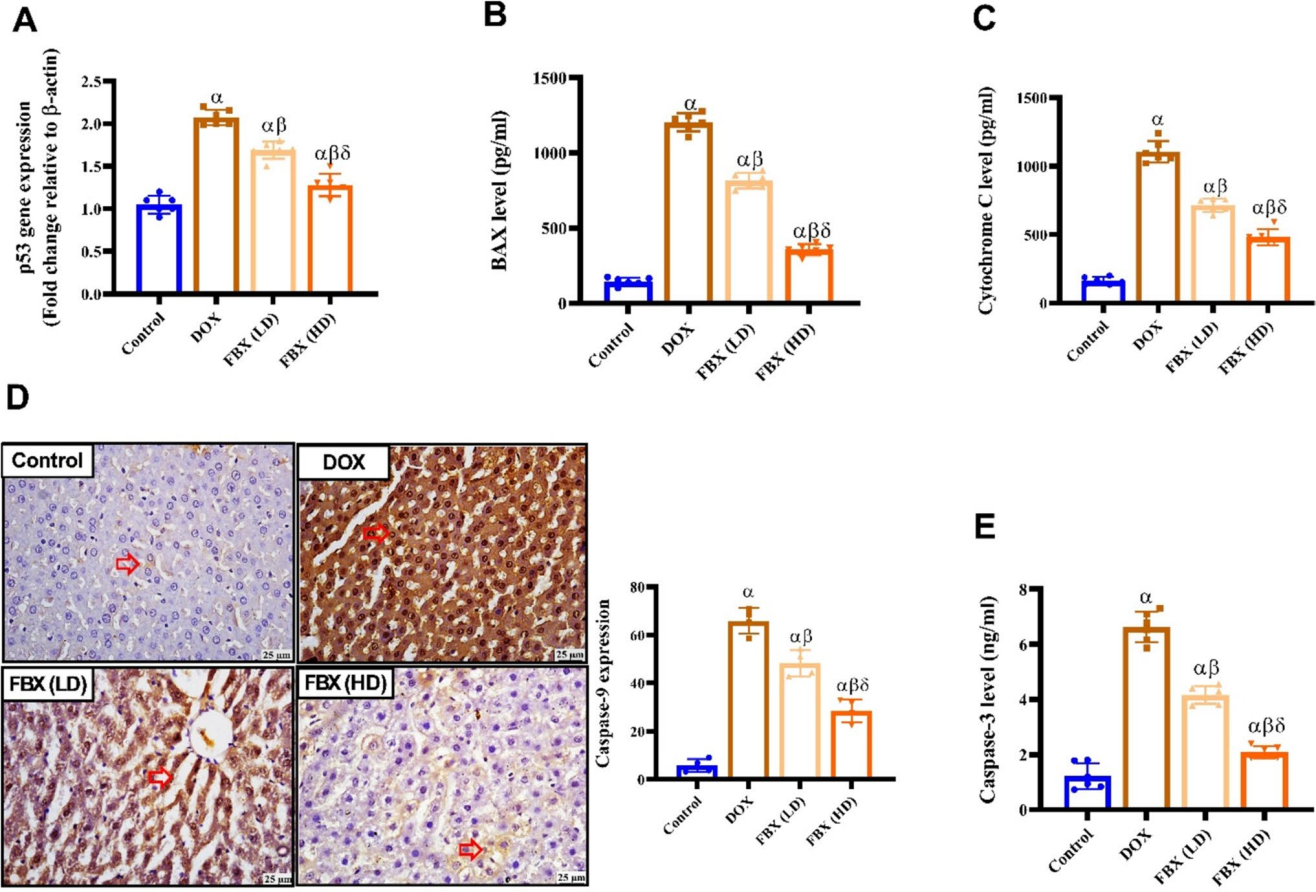
FBX attenuates apoptosis cascades in DOX-administered rats. The anti-apoptotic effect of FBX was evaluated in all groups by measuring; **A** Relative gene expression of TP53 by qRT-PC. **B** hepatic BAX level (pg/ml) by ELISA. **C** hepatic cytochrome C level (pg/ml) by ELISA. **D** Caspase-9 expression by immunohistochemistry. **E** Hepatic active caspase-3 level (ng/ml) by ELISA. Red arrows point to positive expression. IHC counterstained with Mayer's hematoxylin. Bars represent Mean ± SE, (*n* = 4). α, β, δ indicate significance between the control group, DOX group and FBX (LD) treated group respectively using ONE WAY ANOVA test at a *P*-value < 0.05 followed by Tukey

As shown in Fig. 8. expression of P53 and caspase-9 was significantly increased (*p* < 0.05) in DOX group by 0.38 &10.44 folds compared with control group respectively. However, treatment with FBX. in low and high doses significantly decreased (*p* < 0.05) P53 expression by 0.18 and 0.38 folds and expression of caspase-9 content by 0.27 and 0.57 folds respectively compared with DOX group. Furthermore, in relation to the group pre-treated with FBX. (LD10 mg/kg), the group pre-treated with FBX. (HD 15 mg/kg) demonstrated significant further decrease in P53 and caspase-9 expression by 0.24 and 0.41 folds respectively.

Furthermore, content of cytochrome C, caspase-3, BAX was significantly increased (*p* < 0.05) in DOX group by 5.8, 3.63, 7.3 folds compared with control group respectively. However, treatment with FBX. in low and high doses significantly decreased (*p* < 0.05) cytochrome C content by 0.35 and 0.56 folds, caspase-3 content by 0.37 and 0.68 folds and BAX content by 0.32 and 0.7 folds respectively compared with DOX group. Furthermore, in relation to the group pre-treated with FBX. (LD10 mg/kg), the group pre-treated with FBX. (HD 15 mg/kg) demonstrated significant further decrease in Cytochrome C, caspase-3, BAX content by 0.33, 0.5, and 0.56 folds respectively (Fig. 8.).

## Discussion

DOX is a commonly used and extremely efficient antineoplastic drug. However, it has severe multi-organ toxicity (Smuder 2019). The hepatic metabolism of DOX is combined with the ROS production, inflammation and mitochondrial dysfunction (Wali et al. 2020). This promotes cellular apoptosis and decreases the ability of hepatocytes to carry out energy-dependent processes necessary for detoxification and elimination of DOX (Tacar et al. 2013). Therefore, optimizing the therapeutic use of chemotherapeutic agents while reducing their side effects is very desirable. As a result, studies have been conducted to investigate the usage of several agents in combination with DOX to overcome its harmful effects (Asaad et al. 2021; Wu et al. 2021).

Hepatic cell death leads to the enzymes leakage into circulation, and thus, liver dysfunction can be evaluated by the quantification of these biomarkers (Timm et al. 2021). Particularly, elevated circulating AST and ALT are indicators of hepatic damage (Pippa et al. 2020). Although liver impairment is demonstrated in both preclinical and clinical assessments of DOX toxicity, there is currently no treatment to mitigate this negative outcome. In line with prior studies (Sikandar et al. 2020; Abdel Moneim et al. 2023; Negm et al. 2023), this research confirms the hepatotoxic effects of DOX, as demonstrated by raised serum transaminase levels (AST, ALT, and ALP) and histopathological abnormalities within the liver tissue. The rise in liver enzyme levels in the bloodstream is typically indicative of hepatocellular damage and congestion within the central veins and sinusoids, a common consequence of DOX administration (Ijaz et al. 2023). In contrast, the administration FBX has shown a protective effect on the liver, mitigating hepatotoxicity induced by DOX. This is reflected in the decreased levels of serum markers and the amelioration of liver tissue damage in histological examinations.

The transcription factor Nrf2 has an important role in promoting antioxidant enzymes such as glutathione and NADPH and reducing pro-oxidants such as heme. In unstressed cells, Nrf2 is constitutively generated, but it is degraded by the Nrf2-specific ubiquitin ligase complex, which is catalyzed by the stress-sensor protein Keap1. Reactive cysteines in Keap1 molecules are adducted by oxidants and electrophiles, allowing them to sense oxidative stress in cells. Since Nrf2 no longer binds to cysteine-modified Keap1, Nrf2 is able to evade degradation and activates a number of genes related to antioxidant defense, including SOD, catalase, heme oxygenase-1 (Ho-1), and glutathione peroxidase (GPx) (Kensler et al. 2007; Vomund et al. 2017; Bellezza et al. 2018; Hennig et al. 2018; Rojo de la Vega et al. 2018; El-Emam et al. 2020). It has been demonstrated that DOX treatment lowers Nrf2's mRNA and protein expressions, which in turn lowers the expression of downstream antioxidant genes and proteins, causing toxicity of liver (Zhang et al. 2017; Kamble and Patil 2018). To confirm the protective effect of FBX, we examined Nrf2, Keap-1 and HO-1 concentration in relation to DOX administration. In line with prior studies (Zhang et al. 2017; Kamble and Patil 2018), the levels of these proteins were found to be significantly lower in the DOX group than in the control, while FBX upregulated Nrf2, Keap-1 and HO-1 concentration compared to the DOX group in both doses with more pronounced protective effect in the high dose of FBX.

Moreover, our findings showed that, comparing to the control group, the DOX. group had significantly lower levels of catalase and SOD in the liver, decreased concentration of NQO-1, alongside increased NOX1 expression, these results are in fair agreement with the published study by Alzokaky et al. (2023) showing that DOX decreased SOD and GSH contents after its administration (Alzokaky et al. 2023). We also revealed that treatment with FBX reversed these changes compared to the DOX group in both doses with more pronounced protective effect in the high dose of FBX.

SIRT1 is a member of the sirtuin family of proteins, having histone deacetylase activity, which is important for cell differentiation and metabolism (Alves-Fernandes and Jasiulionis 2019). SIRT1 possesses an unique capacity to regulate the Keap1/Nrf2/ARE pathway, perhaps by reducing Keap1 expression, which in turn boosts Nrf2's transcriptional activity and increases ARE-binding capacity (Iside et al. 2020). This may be displayed in the increased heme oxygenase 1 expression and activity, with successive inhibition of ROS overproduction and improvement of the harmful effects of oxidative stress (Wu et al. 2021).

Regarding these issues our finding showed that SIRT1 and AMPK expression is markedly downregulated in DOX group while FBX administration resulted in marked increase in their expression in both doses with more pronounced protective effect in the high dose of FBX in agreement with Abdel-Wahab et al. (2023) stating that FBX exhibits anti-inflammatory and antioxidant properties that lower MDA, MPO, TNF-α, IL-1β, IL-6, and NADPH oxidase levels while raising GSH, SOD, and IL-10 levels. Moreover, FBX decreased NLRP3 expression while raising Sirt-1 expression (Abdel-Wahab et al. 2023). Simultaneously, several studies have demonstrated that DOX is responsible for down-regulation of AMPK activity, which might potentially be the pathway leading to down-regulation of SIRT1 expression (Bartlett et al. 2017).

In addition, inflammation is considered one of the major factors of DOX-mediated liver injury. NF-κB is a transcriptional factor that regulates the expression of several inflammatory genes such as those encoding TNF- α (Cortez et al. 2013; Wang et al. 2017; Kamble and Patil 2018; Soltani Hekmat et al. 2021). NF-κB pathway induction is known to have a critical role in the pathophysiology of DOX-mediated inflammation (Sutariya and Saraf 2018). Here, DOX-treated animals showed raised expression of NF-κB p65, concentration of TNF-α and NLRP3 in addition, while FBX pre-treatment reduced these inflammatory mediators.

Apoptosis is also pivotal in the progression of DOX-induced hepatotoxicity, controlled by the balance between pro-apoptotic and anti-apoptotic proteins. BAX, a pro-apoptotic protein, enhances mitochondrial membrane permeability, allowing cytochrome c to escape from the mitochondrial intermembrane space and activate the intrinsic apoptotic pathway (Ali et al. 2021). In this study, DOX administration was associated with notable increases in cleaved-caspase-3 and BAX levels, along with a rise in cytochrome c levels. Pre-treatment with FBX significantly attenuated these alterations in apoptotic proteins caused by DOX.

**Fig. 9:**
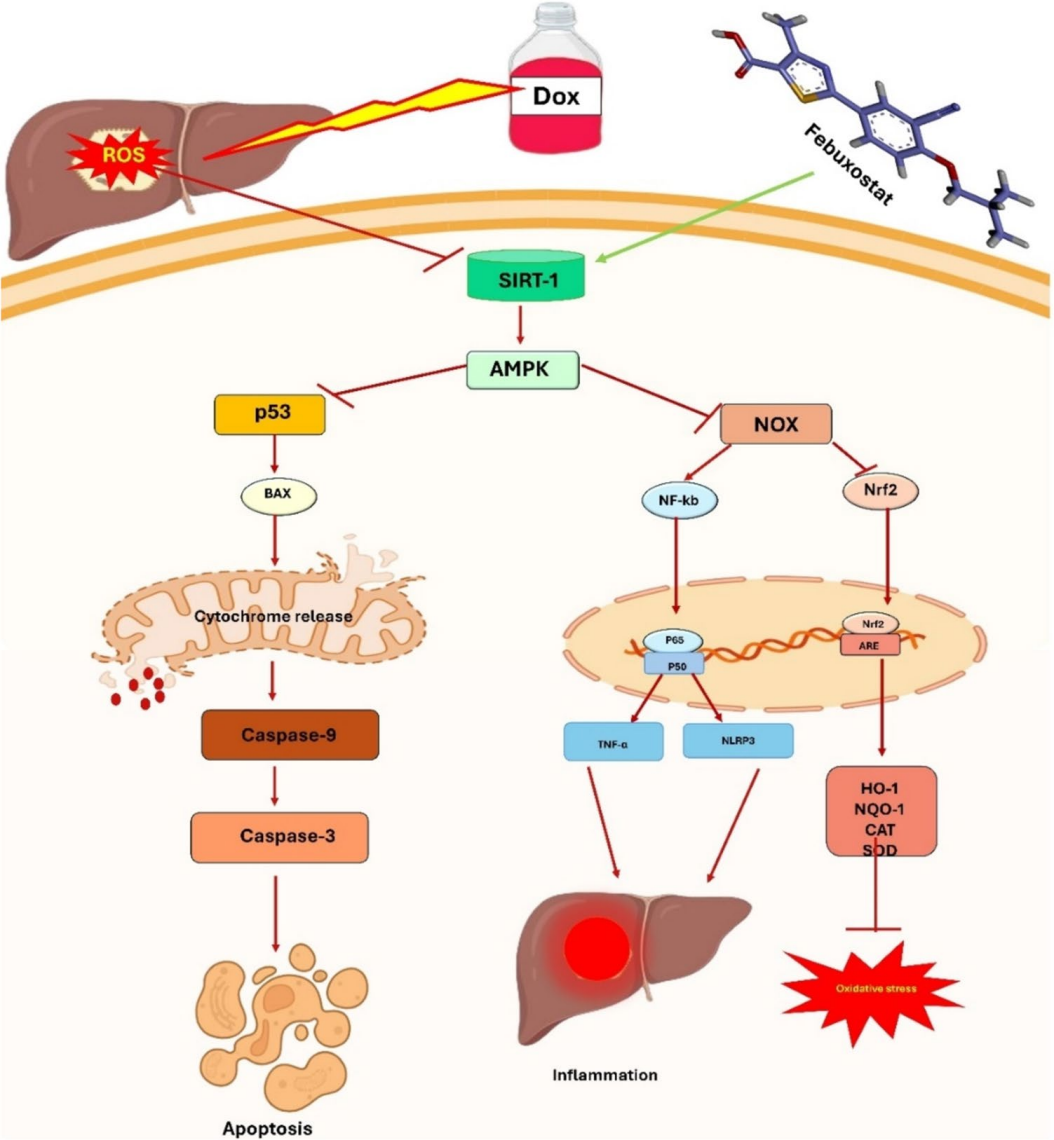
Proposed mechanism of Febuxostat action. Febuxostat alleviates hepatotoxicity induced by doxorubicin in a rat model by activating the SIRT-1/AMPK pathway. This activation influences the transcription or translation of downstream genes crucial for managing oxidative stress, inflammation, and apoptosis

In summary, this research contributes insights into the protective mechanisms of FBX against DOX-induced liver toxicity. FBX mitigated hepatotoxicity and reduced tissue damage. The protective effects of FBX were associated with reduced inflammatory responses and oxidative stress, decreased activation of NF-κB and the inflammasome, enhanced cellular antioxidant defenses, suppression of apoptotic signaling, and the upregulation of the SIRT-1-AMPK anti-inflammatory pathway and Nrf2 signaling, all contributing to its protective efficacy against liver toxicity as illustrated in Fig. 9.

## Limitation of the study

The focus of this study was on doxorubicin-induced hepatotoxicity, which allowed for detailed mechanistic exploration. Future research could extend these findings to other chemotherapeutic agents to determine if the protective effects of febuxostat are generalizable.

The study primarily investigated key oxidative stress, inflammatory, and apoptotic pathways, which are well-documented contributors to hepatotoxicity. Nevertheless, there may be additional pathways influenced by febuxostat that were not explored in this work.

## Conclusion

FBX mitigated DOX-induced hepatotoxicity. The protective effects of FBX were associated with decreased activation of NF-κB and the inflammasome, enhanced cellular antioxidant defenses, suppression of apoptotic signaling, and the upregulation of the SIRT-1-AMPK anti-inflammatory pathway and Nrf2 signaling.

## Data Availability

All source data for this work (or generated in this study) are available upon reasonable request.
